# Supplementary material for: DNA G-quadruplex profiling in skeletal muscle stem cells reveals functional and mechanistic insights
Source: Genome Biol. 2025 Sep 5;26:269. doi: 10.1186/s13059-025-03753-w (PMC12412246; doi:10.1186/s13059-025-03753-w)
Supplement: Supplementary file 1 — Additional file 1: Fig. S1. G4 profiling reveals dynamic remodeling of G4s during mMuSC lineage progression. Fig. S2. G4 profiling reveals dynamic remodeling of G4s during hMuSC activation. Fig. S3. G4s regulate MuSCs function and adult muscle regeneration. Fig. S4. Promoter G4 formation regulates gene transcription in ASCs. Fig. S5. G4s are enriched at loop anchors and promote loop interactions in ASCs. Fig. S6. MAX promotes MuSCs proliferation and adult muscle regeneration. Fig. S7. Uncropped figures for all the western blot results. [file 13059_2025_3753_MOESM1_ESM.zip › Additional file 1_Figure legend.docx]

**Additional file 1: Figure Legends**

**Additional file 1: Fig. S1. G4 profiling reveals dynamic remodeling of G4s during mMuSC lineage progression.** (A) Mouse MuSCs were isolated from PAX7-nGFP mice and FACS gating images are shown. (B) Schematic illustration of G4 CUT&RUN-seq procedure. (C) IF staining of PAX7 (red) in FISCs, PAX7 (red) and MyoD (green) in ASC-48h and MF20 (green) in DSCs to validate the cell identity at each stage during the lineage progression. Nuclei were visualized by DAPI staining (blue). (D) G4 CUT&RUN was performed with three replicates from each of the above stages. Heatmaps showing signal correlation of shared G4 peaks from the three replicates. Pearson correlation coefficient was calculated. (E) Top three motifs enriched in the above identified G4 peaks at different stages. (F) Pie chart showing the distribution of G4 subtypes in the above G4s. (G) Boxplot showing the fraction of PQS from the above identified G4 peaks. (H) Heatmaps showing the PQS and G4 signal enrichment in the above G4 peaks. (I) Heatmap showing the correlation of G4 signals in the four stages. Pearson correlation coefficient was calculated.

**Additional file 1: Fig. S2. G4 profiling reveals dynamic remodeling of G4s during hMuSC activation.** (A) Human MuSCs were isolated by FACS with staining of antibodies (CD31-/CD45-/CD34-/ITGB1+/NCAM+) and the gating images are shown. (B) IF staining of Pax7 (green) to validate the above isolated hFISC cell identity. Nuclei were visualized by DAPI staining (blue). (C) Top three motifs of identified G4 peaks in human MuSCs at different stages. (D) Pie chart showing the distribution of G4 subtypes in the above identified human MuSC G4s. (E) The Boxplot showing the fraction of PQS from the above identified G4 peaks. (F) Heatmaps showing the PQS and G4 signal enrichment in the identified human G4 peaks. (G-H) KEGG pathway analysis of genes with gained promoter G4 in mASC-24h (G) and hASC-24h (H).

**Additional file 1: Fig. S3. G4s regulate MuSCs function and adult muscle regeneration.** (A) ASCs were treated with 5μM PDS or DMSO for 24 or 48 hours and IF stained for γH2AX (green) and DAPI (blue). Representative images are shown. (B) The positively stained cells were quantified from 10 randomly selected fields of three biological replicates. Student’s t test (two-tailed) was used to calculate the statistical significance, n.s., not significant. (C) IF staining figures in Fig. 2G with separate channels.

**Additional file 1: Fig. S4. Promoter G4 formation regulates gene transcription in ASCs**. (A) RNA-seq was performed in mouse FISCs and ASCs. Principal component analysis (PCA) of the variance-stabilized estimated raw counts of differentially expressed genes. Each dot represents an individual biological replicate. (B) Volcano plot showing differentially expressed genes identified in mouse ASC vs. FISC. The red and blue dots denote up- and down-regulated genes, and the green dots denote unchanged genes. (C-D) GO analysis of the above identified up- and down-regulated genes in ASCs. (E) ASCs were treated with PDS or DMSO (control) and RNA-seq was performed. PCA of the variance-stabilized estimated raw counts of differentially expressed genes. Each dot represents an individual biological replicate. (F) Volcano plot showing differentially expressed genes identified in ASCs treated with PDS vs. DMSO. (G) Comparison of DEGs from ASC vs. FISCs and DEGs from ASCs treated with PDS vs. DMSO to identify potential G4 target genes. (H) H3K4me3 signals at the promoter of G4 activated genes in ASCs treated with DMSO or PDS. (I) RNA-seq was performed in hFISCs and hASCs. Scatter plot showing differentially expressed genes in hASCs vs. hFISCs. (J-K) GO analysis of the above identified up- and down-regulated genes. (L-M) KEGG analysis of the enriched pathways of G4-up genes in mASC (L) and hASCs (M).

**Additional file 1: Fig. S5. G4s are enriched at loop anchors and promote loop interactions in ASCs.** (A) Micro-C was performed in ASCs and chromatin loops were identified. Pie chart showing the number of each type of loops. PP: promoter-promoter loop; EE: enhancer-enhancer loop; EP: enhancer-promoter loop; others: none of the above. (B) Schematic illustration of integrative analysis to investigate G4 regulation of chromatin looping in primary myoblast cells integrating the publicly available RNA-seq, Hi-C and H3K27ac ChIP-seq datasets. (C) Loops of different subtypes identified in the above Hi-C data at 5k resolution. (D) G4 signals across the above identified loops. (E) Classification of the above identified loops based on G4 localization at the loop anchors. Pie chart showing the number of each type of G4-related loops. (F) Comparison of interaction frequency of the above different types of G4 loops. Wilcoxon test was used for statistical calculation: n.s., not significant, ***, P < 0.001. (G) The number of genes with promoters associated with each type of G4 loops. (H) Expression level of genes with promoters associated with each type of G4 loops. Wilcoxon test was used for statistical calculation: ***, P < 0.001. (I) mASCs were transfected with two siCCNE1 or control oligos and the knockdown was confirmed by qRT-PCR at 48h post transfection. *18s rRNA* was used as internal control. (J) EdU labeling was performed in the above cells. Representative images are shown. Scale bar = 50 μm. (K) EdU incorporation percentage was quantified from three biological replicates. Student’s t test (two-tailed unpaired) was used to calculate the statistical significance, and p values are shown on the bars. (L) Genomic distribution of MAX binding regions overlapping with G4 peaks in ASCs. (M) Genomic distribution of CTCF binding in primary myoblasts. (N) The number of G4 containing loop anchors with CTCF binding in ASCs. (O) Western blot of MAX and flag in C2C12 cell transfected with control or MAX-flag overexpressing (MAX OE) plasmid. α-Tubulin was used as the normalization control. (P) Co-IP was performed with the Flag antibody in the above cells and the retrieved MAX protein in C2C12 cells transfected with MAX-flag overexpressing plasmid was detected by western blot. (Q) GO analysis of the identified MAX interacting proteins by mass spectrometry.

**Additional file 1: Fig. S6. MAX promotes MuSCs proliferation and adult muscle regeneration.** (A) MAX protein level detected by western blot in ASCs treated with DMSO or PDS. α-Tubulin was used as the normalization control. (B) Schematic of the strategy for MAX inactivation using CRISPR-Cas9 editing in *Pax7^Cas9^* mice. Two sgRNAs were designed to target the exon 1 and exon 2 of MAX ORF, respectively to achieve frameshift mutation of MAX in MuSCs. (C) No obvious morphological difference was observed in Ctrl vs. MAX KD mice. (D) IF staining figures in Fig. 7F with separate channels.

**Additional file 1: Fig. S7. Uncropped figures for all the western blot results.**
