# Supplementary material for: DNA G-quadruplex profiling in skeletal muscle stem cells reveals functional and mechanistic insights
Source: Genome Biol. 2025 Sep 5;26:269. doi: 10.1186/s13059-025-03753-w (PMC12412246; doi:10.1186/s13059-025-03753-w)

Chen X, Yang F and Zhang S et. al. Figure S1

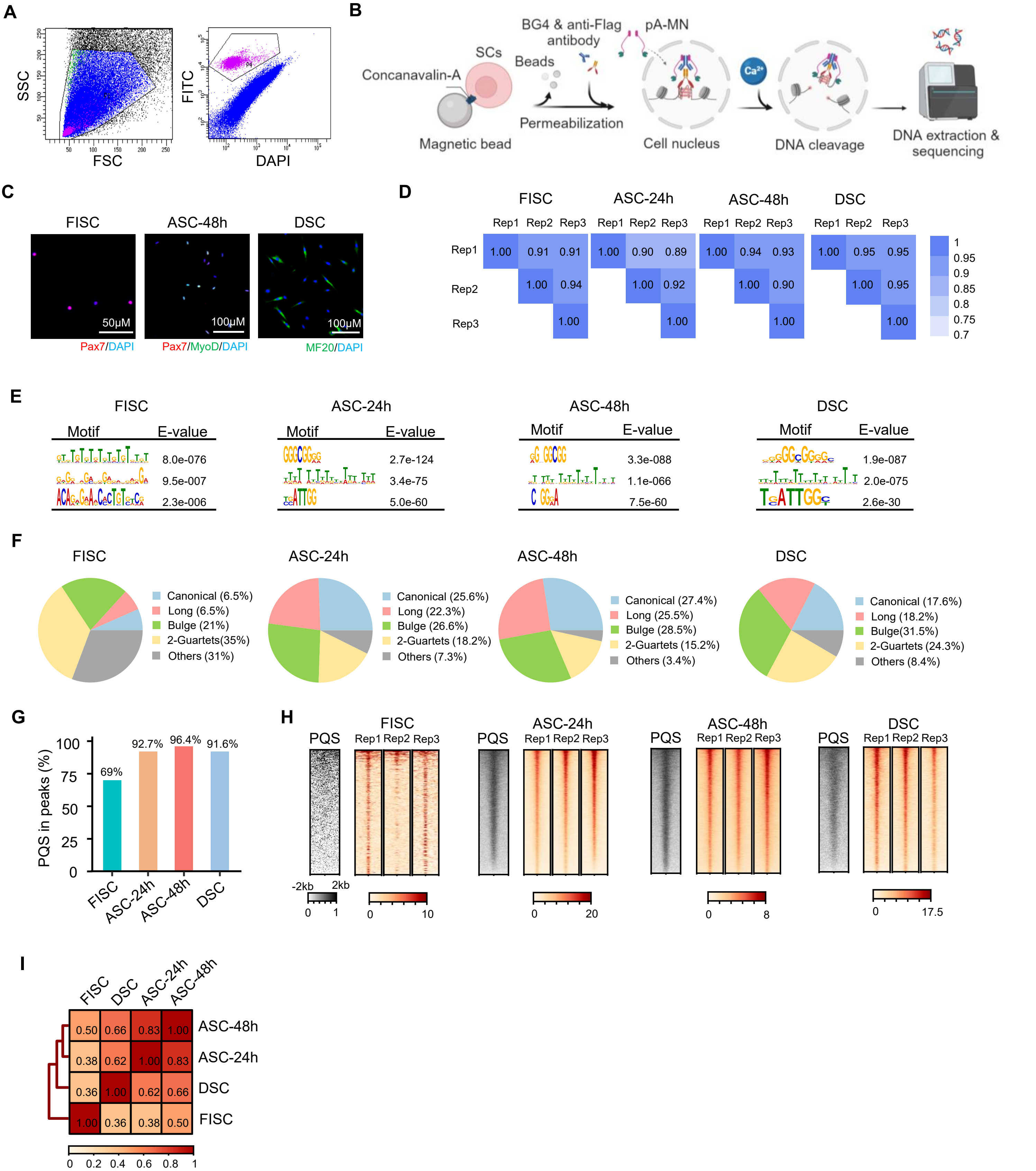

Chen X, Yang F and Zhang S et. al. Figure S2

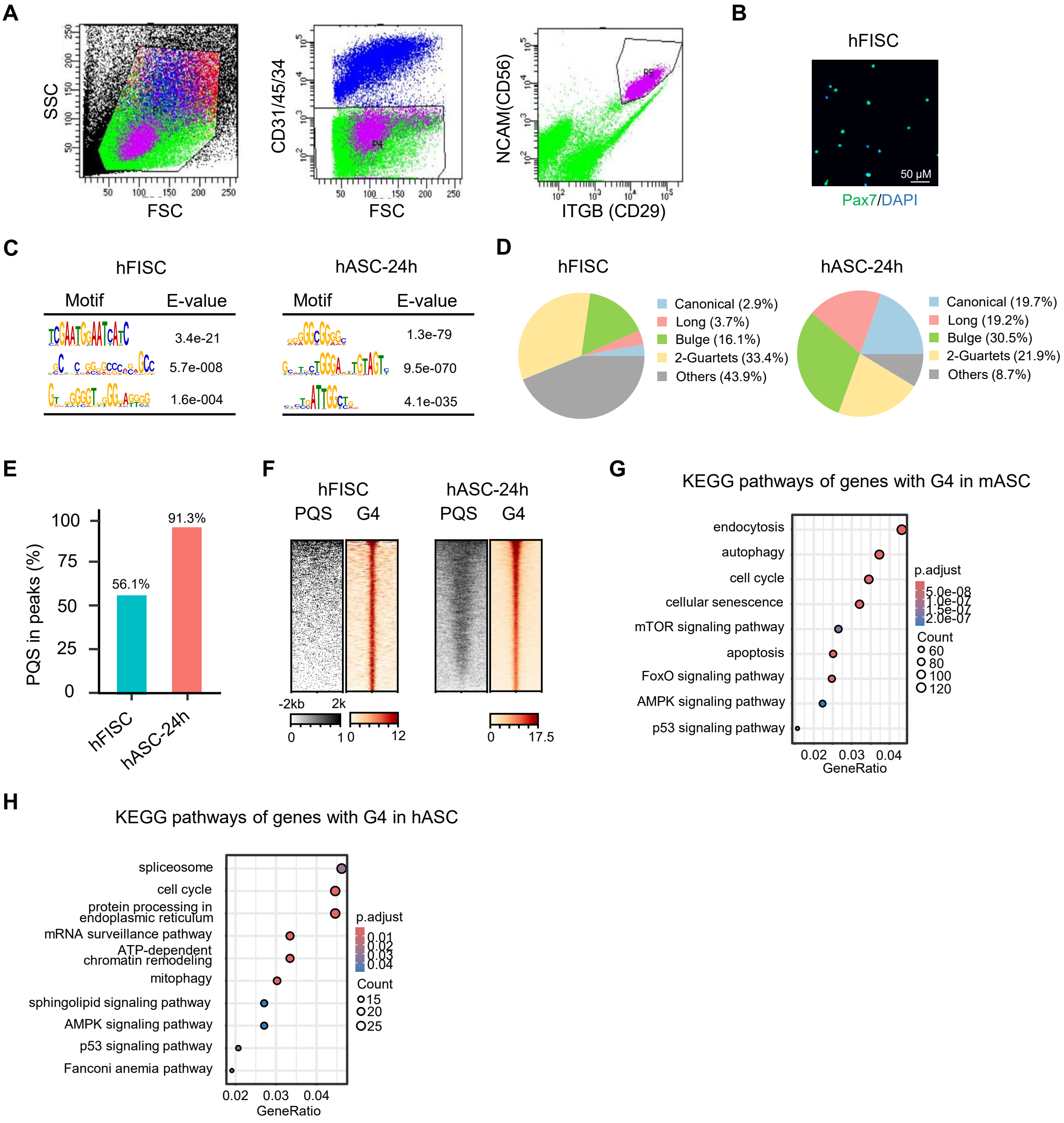

Chen X, Yang F and Zhang S et. al. Figure S3

A

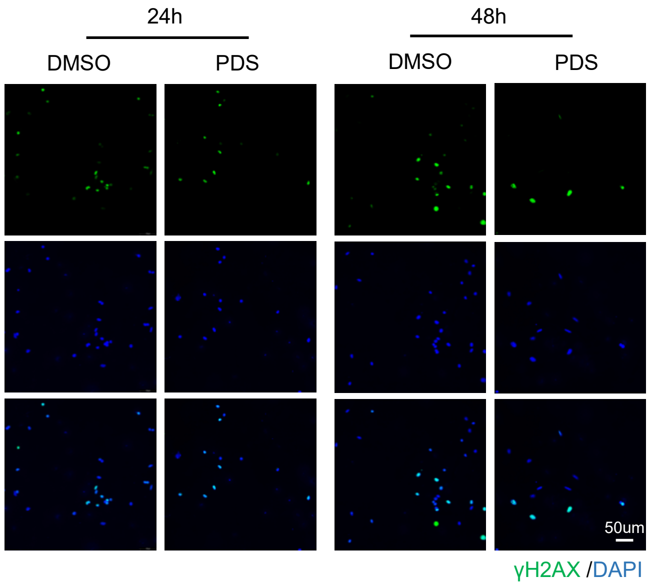

B

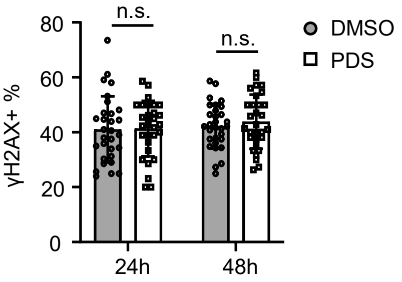

C

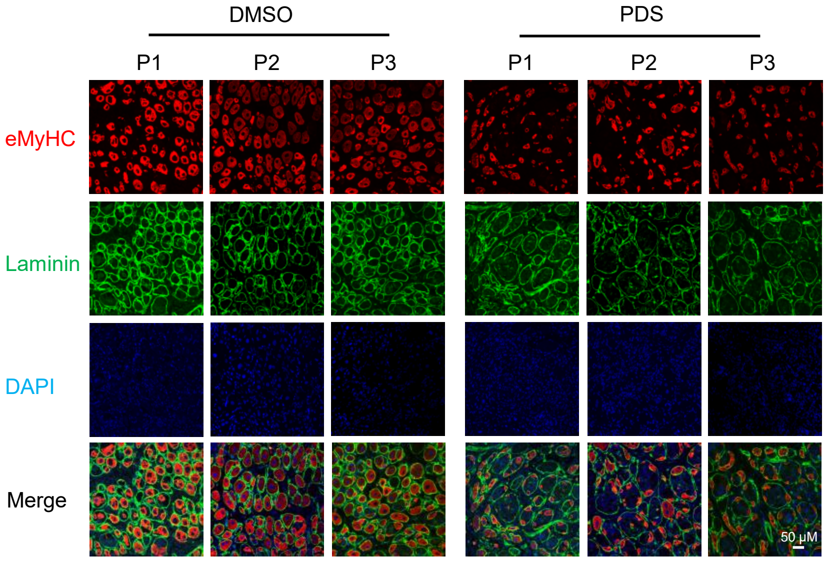

Chen X, Yang F and Zhang S et. al. Figure S4

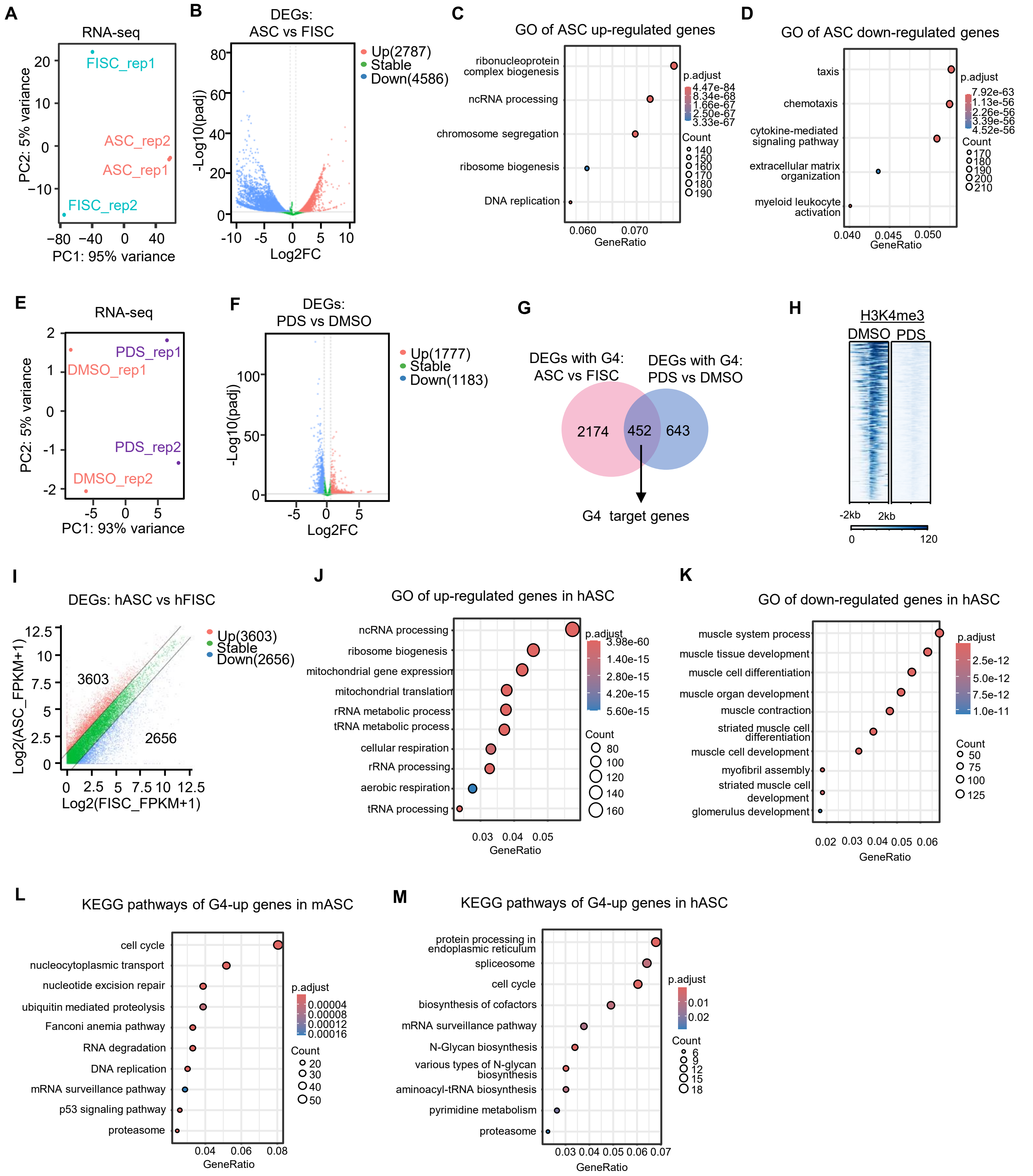

Chen X, Yang F and Zhang S et. al. Figure S5

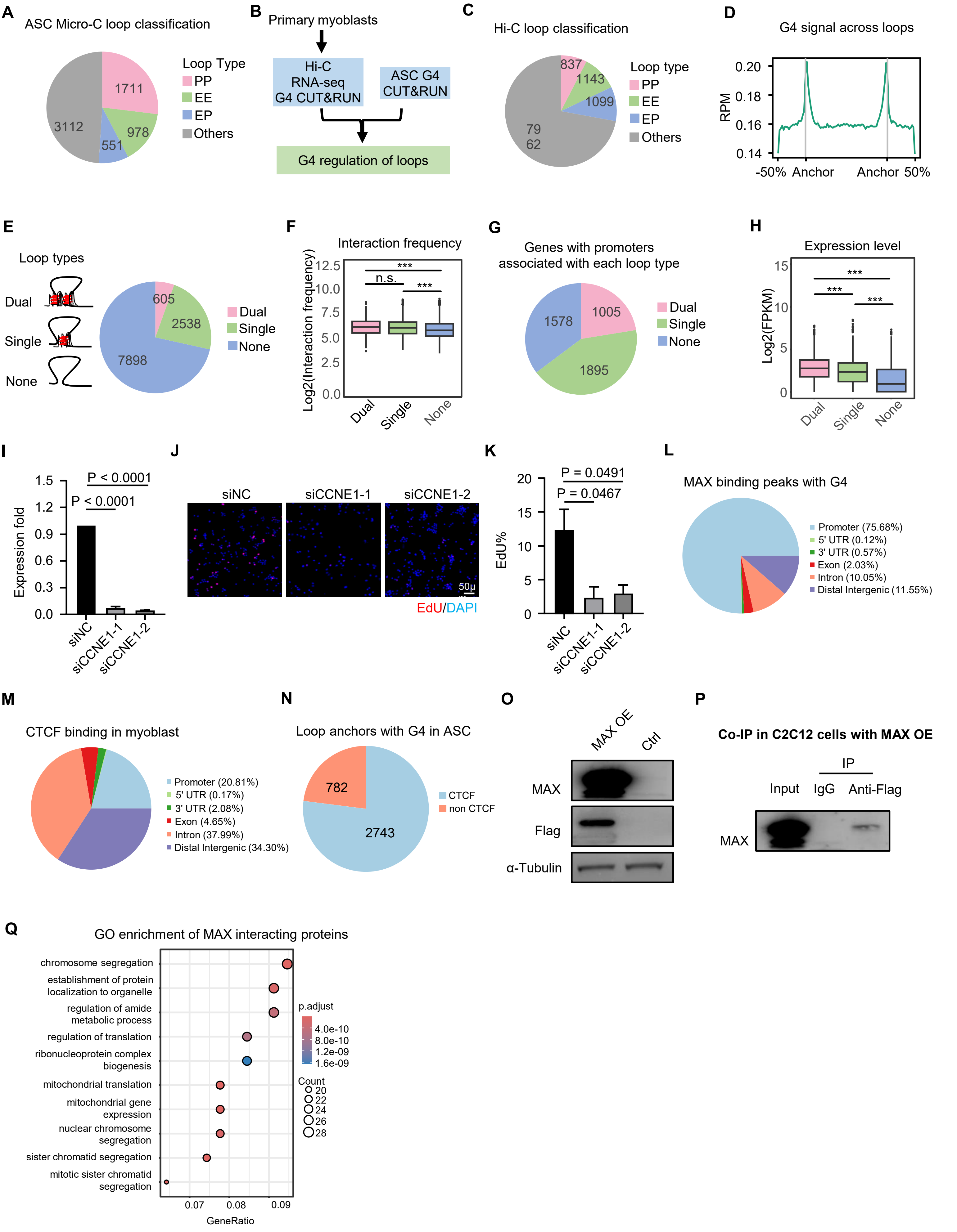

Chen X, Yang F and Zhang S et. al. Figure S6

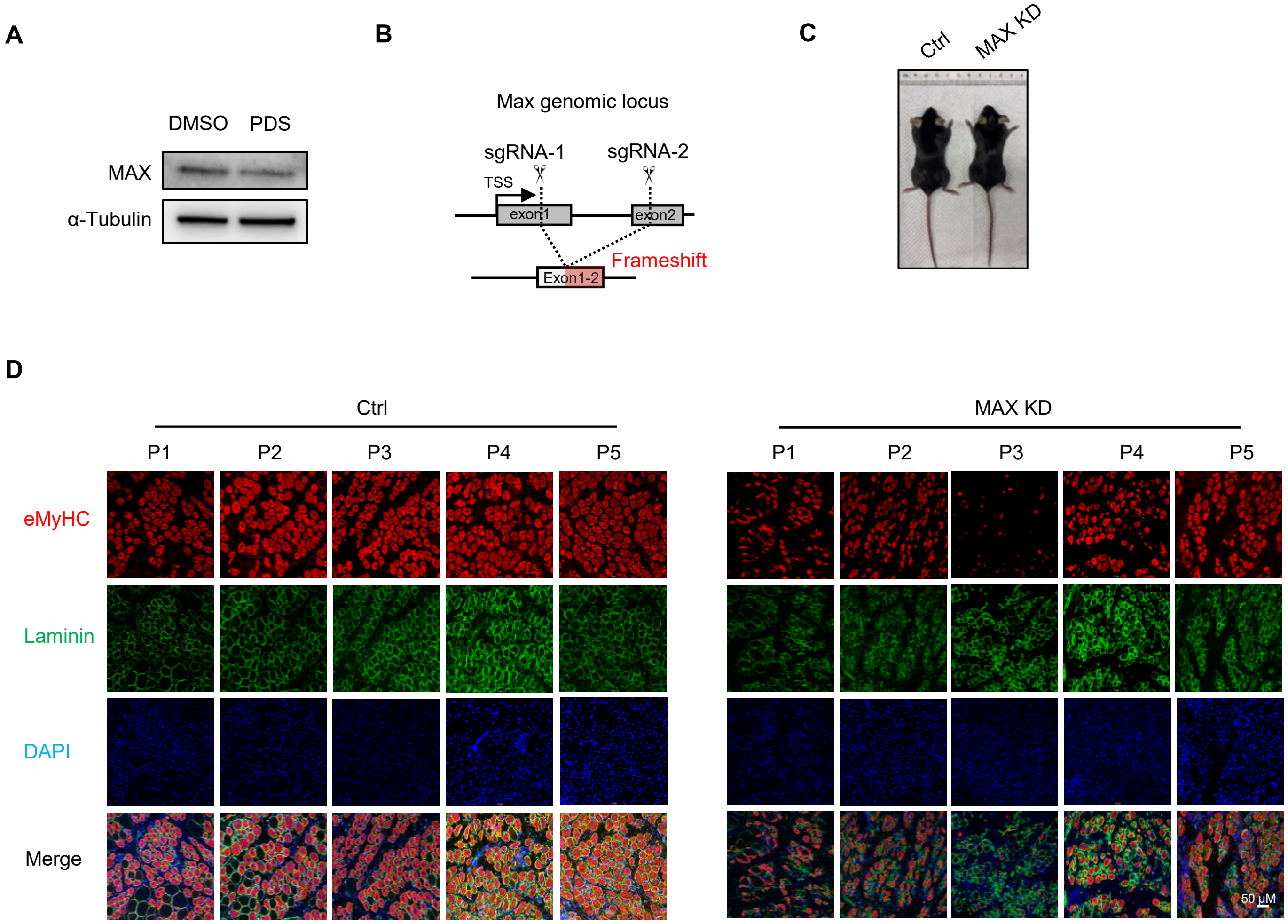

Chen X, Yang F and Zhang S et. al. Figure S7

Uncropped figure for Fig. 6L

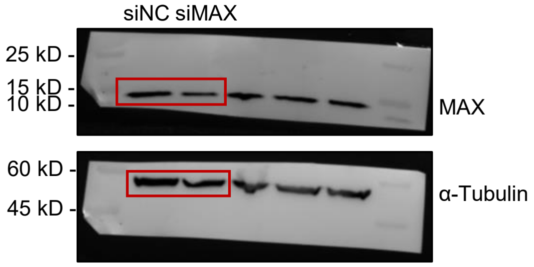

Uncropped figure for Additional file1: Fig. S5O

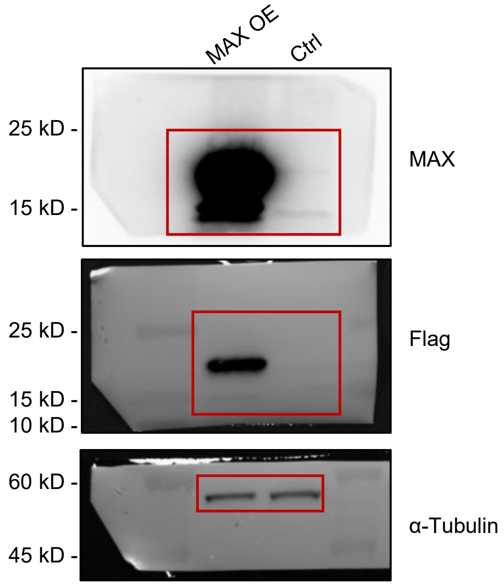

Uncropped figure for Fig. 7A

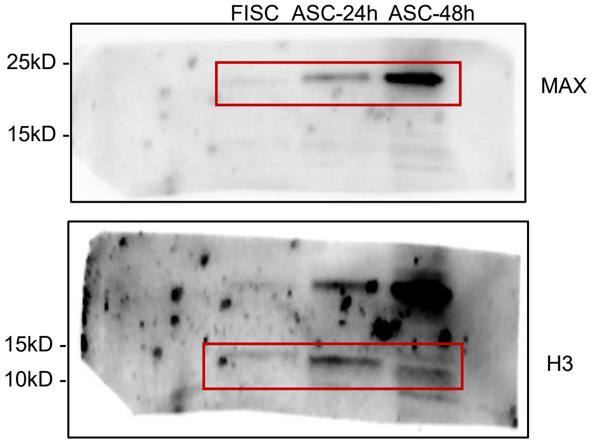

Uncropped figure for Additional file1: Fig. S5P

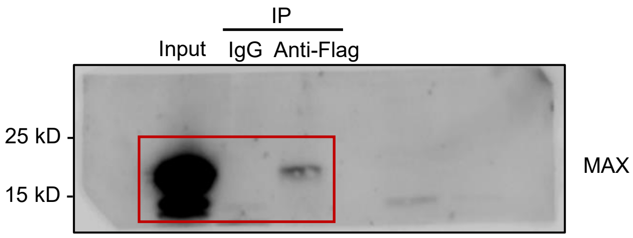

Uncropped figure for Fig. 7D

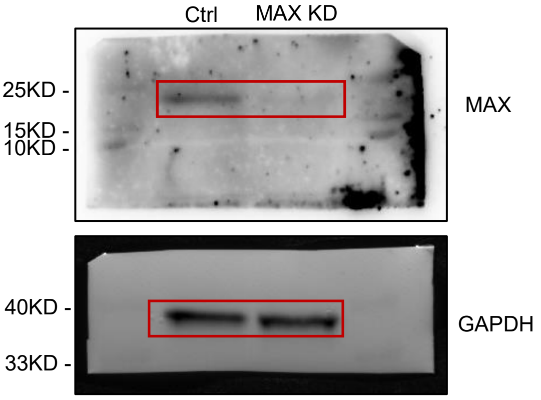

Uncropped figure for Additional file1: Fig. S6A

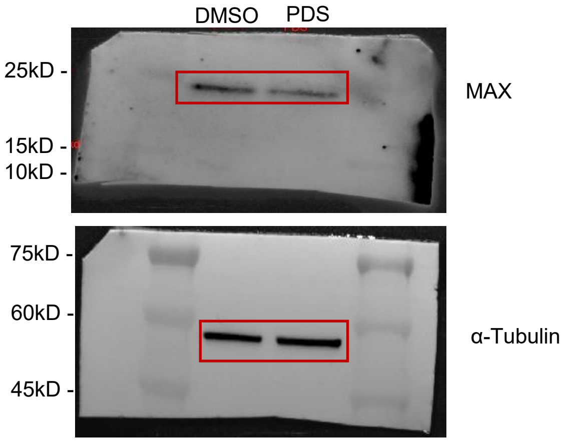

Supplement: Supplementary file 1 — Additional file 1: Fig. S1. G4 profiling reveals dynamic remodeling of G4s during mMuSC lineage progression. Fig. S2. G4 profiling reveals dynamic remodeling of G4s during hMuSC activation. Fig. S3. G4s regulate MuSCs function and adult muscle regeneration. Fig. S4. Promoter G4 formation regulates gene transcription in ASCs. Fig. S5. G4s are enriched at loop anchors and promote loop interactions in ASCs. Fig. S6. MAX promotes MuSCs proliferation and adult muscle regeneration. Fig. S7. Uncropped figures for all the western blot results. [file 13059_2025_3753_MOESM1_ESM.zip › dG4_Supplemental_Figures_RV_3.pdf]
